# Compartmental distribution of GABA_B_ receptor-mediated currents along the somatodendritic axis of hippocampal principal cells

**DOI:** 10.3389/fnsyn.2015.00006

**Published:** 2015-03-23

**Authors:** Claudius E. Degro, Akos Kulik, Sam A. Booker, Imre Vida

**Affiliations:** ^1^Institute for Integrative Neuroanatomy, Neurocure Cluster of Excellence, Charité UniversitätsmedizinBerlin Germany; ^2^Institute for Physiology II, Bioss Centre for Biological Signalling Studies, University of FreiburgFreiburg Germany

**Keywords:** GABA_B_-receptor, Kir3-channel, inhibition, hippocampus, principal cells, dendrites

## Abstract

Activity of cortical principal cells is controlled by the GABAergic system providing inhibition in a compartmentalized manner along their somatodendritic axis. While GABA_A_R-mediated inhibitory synaptic transmission has been extensively characterized in hippocampal principal cells, little is known about the distribution of postsynaptic effects of GABA_B_Rs. In the present study, we have investigated the functional localization of GABA_B_Rs and their effector inwardly rectifying potassium (Kir3) channels by combining electrophysiological recordings in acute rat hippocampal slices, high-resolution immunoelectron microscopic analysis and single cell simulations. Pharmacologically isolated slow inhibitory postsynaptic currents were elicited in the three major hippocampal principal cell types by endogenous GABA released by electrical stimulation, photolysis of caged-GABA, as well as the canonical agonist baclofen, with the highest amplitudes observed in the CA3. Spatially restricted currents were assessed along the axis of principal cells by uncaging GABA in the different hippocampal layers. GABA_B_R-mediated currents were present along the entire somatodendritic axis of principal cells, but non-uniformly distributed: largest currents and the highest conductance densities determined in the simulations were consistently found on the distal apical dendrites. Finally, immunocytochemical localization of GABA_B_Rs and Kir3 channels showed that distributions overlap but their densities diverge, particularly on the basal dendrites of pyramidal cells. GABA_B_Rs current amplitudes and the conductance densities correlated better with Kir3 density, suggesting a bottlenecking effect defined by the effector channel. These data demonstrate a compartmentalized distribution of the GABA_B_R-Kir3 signaling cascade and suggest differential control of synaptic transmission, dendritic integration and synaptic plasticity at afferent pathways onto hippocampal principal cells.

## INTRODUCTION

GABA_B_ receptors mediate the slow inhibitory effects of GABA and contribute crucially to the control of network activity and information processing in cortical circuits by regulating neuronal excitability and synaptic transmission (Kohl and Paulsen, 2010; Palmer et al., 2012; Larkum, 2013). On postsynaptic membranes, GABA_B_Rs preferentially localize to the extrasynaptic membrane and co-cluster with G-protein coupled inwardly rectifying potassium channels (Kir3 or GIRK) (Kulik et al., 2006). Activation of GABA_B_Rs, and subsequent phosphorylation of G_i/o_ leads to activation of Kir3, which produces a slow hyperpolarizing potassium conductance (Bettler et al., 2004). However, the distribution of the receptor and the effector channel is not homogeneous along the somatodendritic axis (Kulik et al., 2003), indicating that this signaling cascade may control excitability of neuronal membranes in a layer- and compartment-specific manner (Larkum, 2013).

Early electrophysiological studies from hippocampal PCs showed that GABA_B_R-mediated slow postsynaptic inhibitory responses can be elicited in the dendrites, but not the perisomatic domain (Newberry and Nicoll, 1985; Solís and Nicoll, 1992). In good agreement, immunocytochemical studies have generally revealed strong labeling in dendritic layers with the highest intensity observed over the distal apical dendrites in the *str. L-M* of the CA areas and the outer ML of the DG (Fritschy et al., 1999; Sloviter et al., 1999; Kulik et al., 2003). Similarly, Kir3 channels were found to show stronger immunolabeling over the distal apical dendrites (Ponce et al., 1996; Drake et al., 1997; Kulik et al., 2006). In stark contrast, recent data from the neocortical layer 5 PCs suggest that perisomatic GABA_B_Rs activate Kir3 potassium channels, whereas dendritic GABA_B_Rs primarily inhibit voltage-sensitive calcium channels (Breton and Stuart, 2012). Whether these contrasting data reflect regional differences in the distribution of GABA_B_Rs and their effectors or a divergent coupling of GABA_B_Rs to their effectors along the somatodendritic axis remains an open question.

In the present study, we therefore analyzed the distribution of GABA_B1_ and Kir3.2 subunits and the currents mediated by GABA_B_R-Kir3 signaling cascade along the somatodendritic axis of hippocampal principal cells in a combined neuroanatomical and electrophysiological approach. Whole-cell patch-clamp recordings were performed *in vitro* from identified principal cells from acute slices and GABA_B_R-Kir3 mediated currents mapped by electrical stimulation, direct pharmacological activation or lamina-specific photolysis of caged-GABA. The distributions of surface membrane localized GABA_B_Rs and Kir3 channels were then assessed with SDS-digested freeze-fracture replica immunogold labeling.

## MATERIALS AND METHODS

### ACUTE SLICE PREPARATION

Experiments were performed on 18–26-days-old Wistar rats, expressing Venus/yellow fluorescence protein (YFP) under the vesicular GABA transporter (vGAT) promoter, (Uematsu et al., 2008) in accordance with local (LaGeSo, Berlin, T 0215/11) and national guidelines (German Animal Welfare Act). Transverse hippocampal slices were prepared as previously described (Booker et al., 2013). Briefly, rats were anesthetized with isoflurane, decapitated and the brains rapidly removed into ice-cold carbogenated (95% O_2_/5% CO_2_) sucrose-modified artificial cerebrospinal fluid (sucrose-ACSF; in mM: 87 NaCl, 2.5 KCl, 25 NaHCO_3_, 1.25 NaH_2_PO_4_, 25 Glucose, 75 Sucrose, 1 Na_2_-Pyruvate, 1 Na_2_-Ascorbate, 7 MgCl_2_, 0.5 CaCl_2_). Transverse hippocampal slices (300 μm nominal thickness) were then cut on a Vibratome (VT1200s, Leica, Germany) in ice-cold sucrose-ACSF, transferred to submerged storage chambers containing sucrose-ACSF warmed to 35°C for 30 min and then stored at room temperature (20°C).

### WHOLE-CELL PATCH-CLAMP RECORDINGS

For electrophysiological recordings, slices were transferred to a submerged recording chamber, and superfused with carbogenated, normal ACSF (in mM: 125 NaCl, 2.5 KCl, 25 NaHCO_3_, 1.25 NaH_2_PO_4_, 25 Glucose, 1 Na_2_-Pyruvate, 1 Na_2_-Ascorbate, 1 MgCl_2_, 2 CaCl_2_), at 10–12 ml/min for improved oxygenation (Hájos et al., 2009) at a near physiological temperature (32 ± 0.4°C) by an inline heater (SuperTech, Switzerland). Slices were visualized with an upright microscope (BX-50, Olympus, Hamburg, Germany) equipped with a 40x water immersion objective lens (N.A. 0.8) and principal cells selected from the *stratum pyramidale* or GCL. Whole-cell patch-clamp recordings were accomplished using a Multiclamp 700B amplifier (Molecular Devices, USA). Recording pipettes were pulled from borosilicate glass capillaries (2 mm outer/1 mm inner diameter, Hilgenberg, Germany) on a horizontal electrode puller (P-97, Sutter Instruments, Novato, CA, USA). When filled with intracellular solution (in mM: 130 K-gluconate, 10 KCl, 2 MgCl_2_, 10 EGTA, 10 HEPES, 2 Na_2_-ATP, 0.3 Na_2_-GTP, 1 Na_2_-creatinine, and 0.1% Biocytin; 290–310 mOsm) the pipettes had a resistance of 3–5 MΩ. Signals were filtered online at 10 kHz using the built in 2-pole Bessel filter of the Multiclamp amplifier, digitized and recorded at 20 kHz (NI USB-6212 BNC, National Instruments, Berkshire, UK), using WinWCP software (courtesy of John Dempster, University of Strathclyde, Glasgow, UK). Data were analyzed oﬄine using the open source Stimfit software package (Guzman et al., 2014^1^).

### CHARACTERIZATION OF GABA_**B**_R-MEDIATED CURRENTS

After achieving whole-cell configuration, intrinsic properties of principal cells were characterized for cell identification. Characterization was performed in current-clamp mode from resting membrane potential (V_m_) and cells identified on the basis of the voltage response and the resulting train of APs of the recorded neurons to a family of hyper- to depolarizing current steps (500 ms duration) ranging from –250 to 250 pA, in 50 pA, steps, followed by a final 500 pA step. Cells showing a hyperpolarized membrane potential, large and fast AP kinetics, and an accommodating train of APs at 500 pA depolarization were deemed to be principal cells.

Pharmacologically isolated GABA_B_R-mediated currents were examined in the presence of ionotropic receptor blockers, DNQX (10 μM), DL-APV (50 μM), and gabazine (10 μM) in the perfusing ACSF, under voltage-clamp at –65 mV. Extracellular stimulation was delivered to the apical neuropil via a glass monopolar electrode (patch pipettes filled with 2 M NaCl, pipette resistance = 0.1 MΩ) and GABA_B_R-mediated IPSCs evoked in response to a single stimulus (100 μs duration, 50 V amplitude) or 200 Hz trains of 3, 5, and 10 stimuli. Stimulation electrodes were positioned at the str. *radiatum/L-M* border for recordings from CA1 and CA3, and in the outer third of the ML for the DG. Kinetic properties of GABA_B_R IPSCs were determined from average traces (minimum eight individual traces), where the IPSC amplitude was greater than 5 pA. To assess the whole-cell contingent of GABA_B_R-mediated currents the canonical agonist baclofen (10 μM) was applied to the bath and 5 min steady state whole-cell current recorded. To confirm that the baclofen-induced current was specific to the GABA_B_R, the potent selective GABA_B_R antagonist CGP 55,845 (CGP, 5 μM) was subsequently applied. The currents produced by baclofen and CGP were measured as the difference in the holding current between the 2 min peak response of each pharmacological epoch and the control level (measured 2 min prior to drug wash-in).

Spatially restricted GABA application was achieved by photolysis of the photolabile caged-GABA compound Rubi-GABA (20 μM) applied to the bath (Rial Verde et al., 2008). Photorelease of GABA was induced by brief flashes (200 ms flash duration, 2 min inter-flash interval) of 470 nm monochromatic light to the tissue (OptiLED, Cairn Scientific, Kent, UK). To assess the spatial distribution of pharmacologically isolated GABA_B_R-mediated currents in hippocampal principal cells, 60 μM wide stripes were exposed to the light flashes in each hippocampal layer, perpendicular to the dendritic axis by fitting a 2 mm slit mask at the level of the stop-filter in the conjugated plan of the epifluorescent tube of the microscope.

### VISUALIZATION, IMAGING, AND RECONSTRUCTION OF THE RECORDED NEURONS

Following completion of the experiments, the outside-out patch configuration was obtained and slices fixed immediately with 4% paraformaldehyde (PFA) in 0.1 M phosphate buffer (PB), overnight at 4°C. Slices were then rinsed repeatedly in PB prior to incubation with Alexa Fluor 647-conjugated streptavidin (1:1000, Invitrogen, Dunfermline, UK), diluted in PB containing 0.1% Triton-X100 and 0.05% NaN_3_, overnight at 4°C. Slices were then rinsed liberally with PB and mounted on glass slides, containing a 300 μm thick agar spacer to reduce compression and shrinkage of the slices, with a polymerizing mounting medium (Fluoromount-G, Southern Biotech, Birmingham, AL, USA) and coverslipped.

Recorded cells were imaged on a laser scanning confocal microscope (FluoView 1000, Olympus) with either 20x (N.A 0.75) or oil-immersion 60x (N.A 1.3) objective lenses for cell identification and reconstructions, respectively. For 3D reconstruction of imaged cells, image stacks were collected from the Z-axis of the cells (0.5 or 1 μm steps, 4 μs pixel dwell time, 4 megapixel resolution). Z-series images for high magnification reconstruction were deconvolved (AutoQuant X3, Media Cybernetics, USA) and stitched using the FIJI software package^2^. Neurons were then segmented and reconstructed using a semi-automatic algorithm in a two-step procedure first tracing the skeleton of the neuron and subsequently fitting the diameters of neurites (Simple Neurite Tracer plug-in for FIJI; Longair et al., 2011). The soma shape was reconstructed by defining the longest axis first and measuring diameters along this path. Morphometric parameters, such as dendritic length and surface area values were derived from the vectorial representation of the reconstructed neurons in the Neuron simulation environment (see below) using predefined morphometric functions (arc3d, diam3d, L and area). Dendritic length and membrane surface area within the illuminated regions were estimated by projecting 60 μm horizontal slits onto the reconstructed neurons and summing the length and area of all segments falling into this region. All light slits were positioned relative to the center of the cell soma; thus a good correlation of light slit position and dendritic length could be produced oﬄine. The surface area of the soma was added to the appropriate slit, but its length was not considered.

### SINGLE CELL SIMULATIONS

The reconstructed neurons were exported from FIJI software using the built in converter to the standard SWC file format for vectorial representation of neuronal structures and imported to the Neuron simulation environment (Hines and Carnevale, 1997; version 7.3 on a Debian Linux PC) using the ‘*import3d*’ tool package. All neurons were rotated to a vertical position with their somatodendritic axis, to match their orientation during the experiments. Scales were checked and Z axis dimensions corrected by measuring the embedded slice thickness and using a correction factor assuming an original slice thickness of 300 μm. To reduce raggedness of reconstructed neuronal process trajectories, in particular along the Z-axis, a Gaussian spatial filter was applied (five point window, single run in the X–Y plane and 10 iterations for values along the Z-axis). Diameters were checked for non-fitted values which were present in the SWC files and these were substituted by linear interpolation to neighboring points.

The electrical behavior of the neuron was assumed to be passive. The specific membrane capacitance (C_m_) was set to 1 μF/cm^2^, and the axial resistivity (Ri) was 140 Ohm/cm (Baker et al., 2011) for all cells, The resting membrane potential and the reversal of the leak conductance (*pas* distributed mechanism) were set to –65 mV. Passive membrane resistivity (R_m_) in the default model was assumed to be non-uniform for all PCs, with 50% lower values at the distal dendrites along a sigmoidal gradient (Stuart and Spruston, 1998; Golding et al., 2005):

$$R_{m}=R_{m}{\left(soma\right)}^{*}\left(0.5+0.5/\left(1+Exp(D_{x}-D_{half})/f_{steepness}\right)\right)$$

where D_x_ is the distance of a given point measured from the center of the soma, the gradient midpoint D_half_ was 150 μm from the soma and the steepness factor f_steepness_ was 50 μm. For granule cells a uniform value was applied to the entire dendritic axis (Schmidt-Hieber et al., 2007). The value of R_m_ was then individually determined for each cell by matching the measured input resistance.

Spines were not reconstructed but were incorporated in the model as a surface area correction factor: the extra area contributed by the spines was modeled by dividing Rm and multiplying Cm by a dendritic domain specific factor (Stuart and Spruston, 1998; Golding et al., 2005). These correction factors were based on electron microscopic spine density measurements by Megías et al. (2001) and confirmed and derived for simulations in the study by Golding et al. (2005). The correction factors were between 1.0 (soma and proximal dendrites largely lacking spines) and 3.3 (high density spines on thin oblique dendrites in the CA1 *str. radiatum*). In the modeled CA1 and CA3 PCs spines contributed to an average of 57 and 49% of the total membrane area, respectively. In granule cells spine correction factors approximated the distribution described by Schmidt-Hieber et al. (2007) and had a value of 1.7 for the proximal dendritic segments in the inner ML, 2.1 for those in the middle and 2.3 for segments in the outer ML.

After passive electric properties were applied, segment length was adjusted according to the “d_lambda rule” (Carnevale and Hines, 2009): an alternating current length constant at 1 kHz was calculated for each section, and the number of segments per dendritic section (*nseg*) was increased until their length was less then 3.3% of this length constant (Schmidt-Hieber et al., 2007). The integration time step was fixed to 12.5 μs. Voltage-clamp recordings were simulated with a *VClamp* object positioned at the soma, with the electrode resistance set to the value of the uncompensated series resistance during the experiment and the holding potential was –65 mV.

GABA_B_ receptor-mediated synaptic effects were modeled by inserting *Exp2Syn* point processes into the segments falling into the illumination windows. Peak conductance was calculated by multiplying the assumed current density with the surface area of the corresponding segment located within the illumination window. Kinetic parameters of the conductance were set to the experimentally determined values and the reversal potential was –95 mV (Booker et al., 2013). All instances of the synaptic conductance inserted into a cell were connected to and triggered by an abstract presynaptic *NetStim* object. For each simulation run the peak amplitude of the somatically measured GABA_B_R-mediated current was measured by recording the current measured by the VClamp object and the conductance density for the illumination window was iteratively adjusted on proportion to the error (the difference between the experimentally measured amplitude and the peak value of the somatic current obtained in a simulation run) until the difference was smaller than 0.01 pA. For each window the conductance density was calculated as the mean of at least three iterative search processes. Initial values were randomly chosen from a uniform distribution with 25% variability around the current density calculated as the ratio of the somatically measured current divided by the driving force and the surface area within a given illumination slit.

### SDS-DIGESTED FREEZE-FRACTURE REPLICA IMMUNOGOLD LABELING

To assess lamina distribution of the GABA_B1_ and Kir3.2 subunits SDS-FRL was performed as previously described (Kulik et al., 2006). Transgenic vGAT Venus/YFP mice (30-days-old; *n* = 3) were lightly anesthetized with isoflurane; followed by terminal anesthesia with ketamine/Domitor (5:3 mix, 6.3 and 0.8 mg/kg respectively, i.p.). The rats were then transcardially perfused with 0.9% NaCl for 1 min, followed by fixative solution containing 2% PFA and 15% saturated picric acid (in PB), for 13 min. Transverse hippocampal sections (90 μm) were cut on a vibratome (VT1000, Leica, Germany) and cryoprotected overnight with 30% glycerol in PB, at 4°C. Blocks containing either CA1 and DG or CA3 were microdissected from the sections and frozen under high-pressure (HPM100, Leica, Germany). Frozen samples were fractured at –130°C and the fractured face coated by deposition of carbon (5 nm), platinum (2 nm) then carbon (18 nm) in a freeze-fracture replica machine (BAF060, BAL-TEC, Lichtenstein). Replicas were digested for 18 hrs at 80°C in a solution containing 2.5% SDS and 20% sucrose diluted in 25 mM Tris buffered saline (TBS), pH 7.4. Following digestion, replicas were washed liberally in replica washing solution, which contained 0.05% bovine serum albumin (BSA) and 0.1% Tween 20, in TBS; then blocked in a solution containing 5% BSA and 0.1% Tween 20 for 1 h at room temperature. Replicas were then incubated with primary antibodies raised against either the GABA_B1_ subunit (B17, rabbit, 10 μg ml^-1^; Kulik et al., 2003, 2006; Booker et al., 2013) or Kir3.2 subunit (rabbit, 8 μg ml^-1^, Alomone Labs, Israel), in a solution containing 1% BSA and 0.1% Tween 20 made up in TBS, overnight at 4°C. The replicas were then washed liberally in TBS, blocked for 30 min and then reacted with 10 nm gold nanoparticles conjugated to goat anti-rabbit secondary antibodies (1:30, Nanoprobes, Yaphank, NY, USA) diluted in a solution containing 1% BSA and 0.1% Tween 20 made up in TBS, either for 3 h at room temperature or overnight at 4°C. Replicas were washed in TBS, then ultrapure water and mounted on 25-mesh grids. For quantitative analysis, replicas were first imaged with light microscopy to determine laminar organization and then images of P-face spiny dendrites or somata were collected from the middle portion of the layers of CA1, CA3, and DG. Immunogold particle density was calculated by analyzing the number of immunogold particles on the total exposed P-face surface of the somatic or dendritic membrane using FIJI/ImageJ software package.

### CHEMICALS AND PHARMACOLOGICAL TOOLS

Chemicals were obtained from either Sigma Aldrich (Munich, Germany) or Carl Roth (Karlsruhe, Germany). Biocytin was obtained from Life Technologies (Dunfermline, UK). Drugs were obtained from Abcam Biochemicals (Cambridge, UK) or Tocris Bioscience (Bristol, UK). Drugs were stored as 1000-fold concentrated stocks at –80°C. Working concentrations were prepared fresh on the day in normal ACSF: DNQX 10 μM, DL-APV 50 μM, gabazine (SR-95531)10 μM, Rubi-GABA 20 μM, CGP-55845 5 μM, and baclofen 10 μM.

### STATISTICAL ANALYSIS

Statistical analysis was performed with Graphpad Prism 3.0 (GraphPad Software, La Jolla, CA, USA). Group data were compared with either one-way ANOVA (parametric analysis) or Friedman (non-parametric) tests, respectively combined with Bonferroni or Dunn’s multiple comparison post-test to establish group differences. Analysis of unpaired and paired data was performed with Mann–Whitney or Wilcoxon matched-pairs tests respectively. Data is shown as mean ± SEM throughout. Statistical significance was assumed if *P* < 0.05.

## RESULTS

### POSTSYNAPTIC GABA_**B**_R CURRENTS SHOW DIFFERENTIAL AMPLITUDES IN CA1, CA3 PCs, and DGCs

**FIGURE 1:**
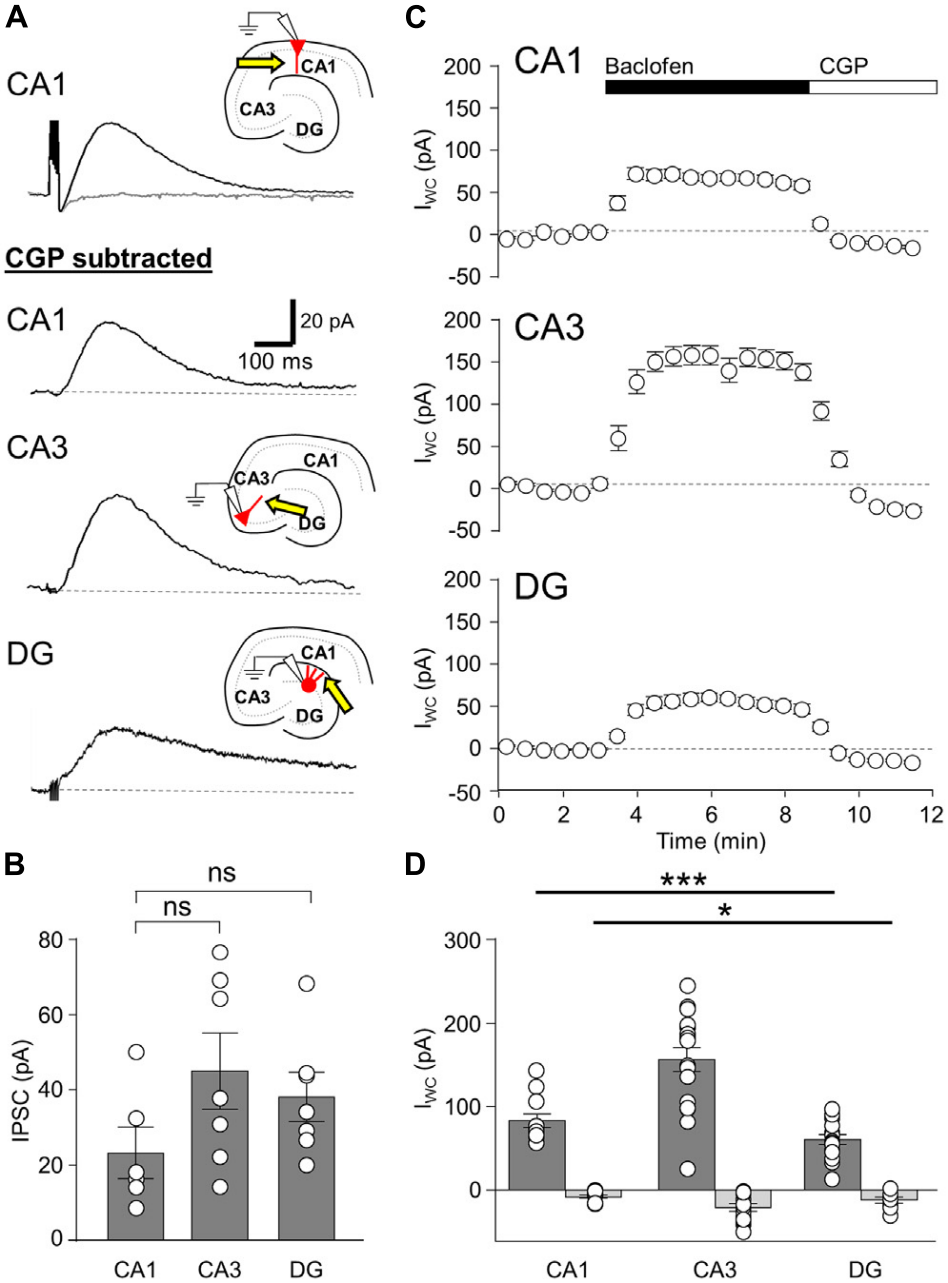
**Synaptic and agonist-induced GABA_**B**_R-mediated currents in hippocampal principal cells.** (**A**, upper), IPSCs elicited in a CA1 PC by a train of five stimuli at 200 Hz before (black trace) and after application of the specific GABA_B_R-antagonist CGP-55845 (CGP, 5 μm, gray trace). (**A**, lower), IPSCs elicited by a train of five stimuli in CA1 PCs (top), CA3 PCs (middle) and DGCs (bottom), following subtraction of the CGP trace. Inset illustrates the arrangement of the recording pipette and the extracellular stimulation site (arrow). **(B)** Summary bar chart shows the mean amplitudes of GABA_B_R-mediated IPSCs produced by 5-stimuli in CA1 PCs (CA1, *n* = 6), CA3 PCs (CA3, *n* = 7), and DGCs (DG, *n* = 7). **(C)** Mean change in I_WC_ plotted against time, before and during baclofen and subsequent CGP application (CA1 PCs, *n* = 5; CA3 PCs, *n* = 9; DGCs, *n* = 8). **(D)** Bar chart of the average peak I_WC_ measured for baclofen (dark gray) and CGP (light gray) in CA1 PCs (baclofen: *n* = 12, CGP: *n* = 11), CA3 PCs (baclofen: *n* = 17, CGP: *n* = 13), and DGCs (baclofen: *n* = 15, CGP: *n* = 9). All average data is overlain by the values from the individual experiments (open circles). Statistics shown: ns = *P* > 0.05, *P < 0.05, ***P < 0.001; one-way ANOVA.

Previously reports have observed a clear laminar staining pattern for the GABA_B1_ subunit of the obligatory heterodimer receptor and the Kir3.2 channel subunit in the hippocampal neuropil (Fritschy et al., 1999; Sloviter et al., 1999; Kulik et al., 2003, 2006). To confirm the presence of GABA_B_R/Kir3-mediated potassium currents in hippocampal principal cells (Dutar and Nicoll, 1988; Solís and Nicoll, 1992; Lüscher et al., 1997; Mott et al., 1999; Booker et al., 2013) we performed extracellular stimulation of pharmacologically isolated GABA_B_R-mediated slow IPSCs and tested the response of principal cells to the canonical GABA_B_R agonist baclofen. To assess GABA_B_R-mediated responses produced by synaptic release of GABA, we elicited slow IPSCs in the presence of AMPA, NMDA, and GABA_A_ receptors antagonists (DNQX, 10 μM; APV, 50 μM and gabazine, 10 μM). Slow IPSCs were evoked by electrical stimulation to the neuropil surrounding the distal apical dendrites with a single stimulus or trains of five stimuli delivered at 200 Hz (**Figure 1A**, top). All recorded IPSCs were confirmed to be GABA_B_R-mediated due to their blockade during bath application of the potent and selective GABA_B_R antagonist CGP-55845 (CGP; 5 μM).

In CA1 PCs (*n* = 5 cells), extracellular stimulation of the *str. radiatum/L-M* border produced IPSCs following a single stimulus with a mean peak amplitude of 7.7 ± 1.2 pA, while trains of the 5-stimuli (applied at 200 Hz) produced IPSCs with a mean amplitude of 26.2 ± 6.8 pA (**Figure 1A**, top and **1B**), consistent with an increased GABA release and therefore greater volume transmission during the trains. In CA3 PCs (*n* = 7 cells), a single stimulus to the *str. radiatum/L-M* border produced IPSCs with a mean amplitude of 12.4 ± 0.8 pA, somewhat larger than those observed in CA1 PCs, albeit not significantly so (*P* = 0.15). The 5 stimulus train elicited IPSCs of 45.0 ± 9.4 pA in CA3 PCs, (**Figures 1A**, middle and **1B**). Finally, slow IPSCs in DGCs (*n* = 6 cells) had mean amplitudes of 8.1 ± 1.2 pA following single stimuli and 39.6 ± 7.0 pA following the 200 Hz trains of five stimuli. (**Figures 1A**, bottom, **1B**).

The kinetics properties of GABA_B_R-mediated IPSCs, measured from single stimulus responses (>5pA) were mostly comparable for the three principal cell types (**Table 1**). IPSCs recorded in CA1 PCs (*n* = 5), CA3 PCs (*n* = 7), and DGCs (*n* = 6) showed similar onset latencies and rise times (*P* > 0.05, one-way ANOVA). Surprisingly however, the decay time-constant of the IPSCs in DGCs was ∼100% longer than either CA1 or CA3 PCs (*P* = 0.04, one-way ANOVA, with Bonferroni multiple comparisons, **Table 1**).

**Table 1:** Amplitude and kinetic properties of GABA_**B**_R mediated IPSCs.

	Amplitude (pA)	Onset latency (ms)	Rise-time (ms)	Decay time constant (ms)
CA1 PC (*n* = 5)	7.7 ± 1.2	51.0 ± 6.9	106.2 ± 13.2	188.6 ± 38.9
CA3 PC (*n* = 7)	12.4 ± 0.8	47.2 ± 2.6	114.0 ± 11.2	223.0 ± 49.5
DGC (*n* = 6)	8.1 ± 1.2	53.5 ± 7.3	86.8 ± 7.0	428.1 ± 89.6**

Kinetic responses of IPSCs produced in response to single extracellular stimulation to the distal dendrites. ** indicates difference from CA1 PCs (*P* < 0.05, Mann–Whitney non-parametric test).

As extracellular stimulation only activates a subset of GABA_B_Rs on the somatodendritic domain of neurons (Lüscher et al., 1997; Mott et al., 1999; Booker et al., 2013), we next bath applied the canonical GABA_B_R agonist baclofen (10 μM) in order to activate the full complement of surface localized functional receptors (in the presence of ionotropic receptor blocker DNQX or NBQX, APV, and gabazine). In CA1 PCs, baclofen application elicited an outward whole-cell current (I_WC_) of 83.1 ± 7.9 pA (*n* = 12 cells, **Figure 1C**, top). In CA3 PCs the observed baclofen-induced peak I_WC_ was substantially larger at 156.3 ± 14.1 pA (*n* = 17 cells) 188% higher than those in CA1 PCs (*P* = 0.0007; **Figure 1C**, middle). In contrast, DGCs responded to bath application of baclofen with a smaller I_WC_ of 60.6 ± 5.9 (*n* = 15 cells, **Figure 1C**, bottom). This was 27% lower than the currents recorded in CA1 PCs (*P* = 0.04) and 61% lower than those in CA3 PCs (*P* < 0.0001, **Figure 1D**).

In all tested principal cells the baclofen-induced I_WC_ was fully blocked by subsequent application of CGP (5 μM), confirming the specificity of the baclofen-induced currents. Moreover, the mean I_WC_ during CGP steady state undershot baseline current levels significantly in all cell types (*P* < 0.01 for all, **Figure 1D**). The mean amplitude of the overshoot current was 7.5 ± 1.9 pA in CA1 PCs (*n* = 11), 20.5 ± 4.6 pA in CA3 PCs (*n* = 13), and 11.7 ± 3.2 pA in DGCs (*n* = 9; **Figures 1C,D**). This observation suggests that a small net GABA_B_R tonic current, mediated by either Kir3 channels or inhibition of calcium channels, was present in the slices prior to baclofen application, contrary to previously published literature (Otis and Mody, 1992).

GABA_B_ receptor-mediated postsynaptic currents are predominantly mediated by activation of Kir3 channels (Otis et al., 1993; Sodickson and Bean, 1996; Lüscher et al., 1997; Booker et al., 2013). We have shown previously, under identical experimental conditions (Booker et al., 2013), that GABA_B_R-mediated currents in CA1 PCs and interneurons have a reversal potential close to –100 mV and display inward-rectification, as typical for Kir3 channels. To confirm that the same is true in other principal cells, we tested the reversal potential (E_GABAB_) of GABA_B_R-mediated IPSPs from current-clamp recordings of DGCs. The observed E_GABAB_ for the GABA_B_R-mediated IPSP was –89.5 ± 3.2 mV (*n* = 3 cells), close to the predicated potassium E_GABAB_ of –101 mV (data not shown). Furthermore, currents observed in response to voltage-ramps from –20 to –120 mV (1 s duration), ran before and after the application of baclofen, showed a K^+^ E_R_ of –98.3 ± 5.8 mV with a rectification index of 0.59 (*n* = 3 cells, data not shown), confirming that baclofen-mediated currents were produced by inwardly rectifying K^+^-channels in DGCs.

In summary, all hippocampal principal cells express GABA_B_R-mediated slow IPSCs, plausibly by activation of Kir3 channels, albeit with cell-type specific differences in the magnitude of the whole-cell conductance indicating potential differences in the postsynaptic complement of the receptor and effector channels.

### LAMINAR DISTRIBUTION OF FUNCTIONAL GABA_**B**_R-MEDIATED CURRENTS IN HIPPOCAMPAL PRINCIPAL CELLS

**FIGURE 2:**
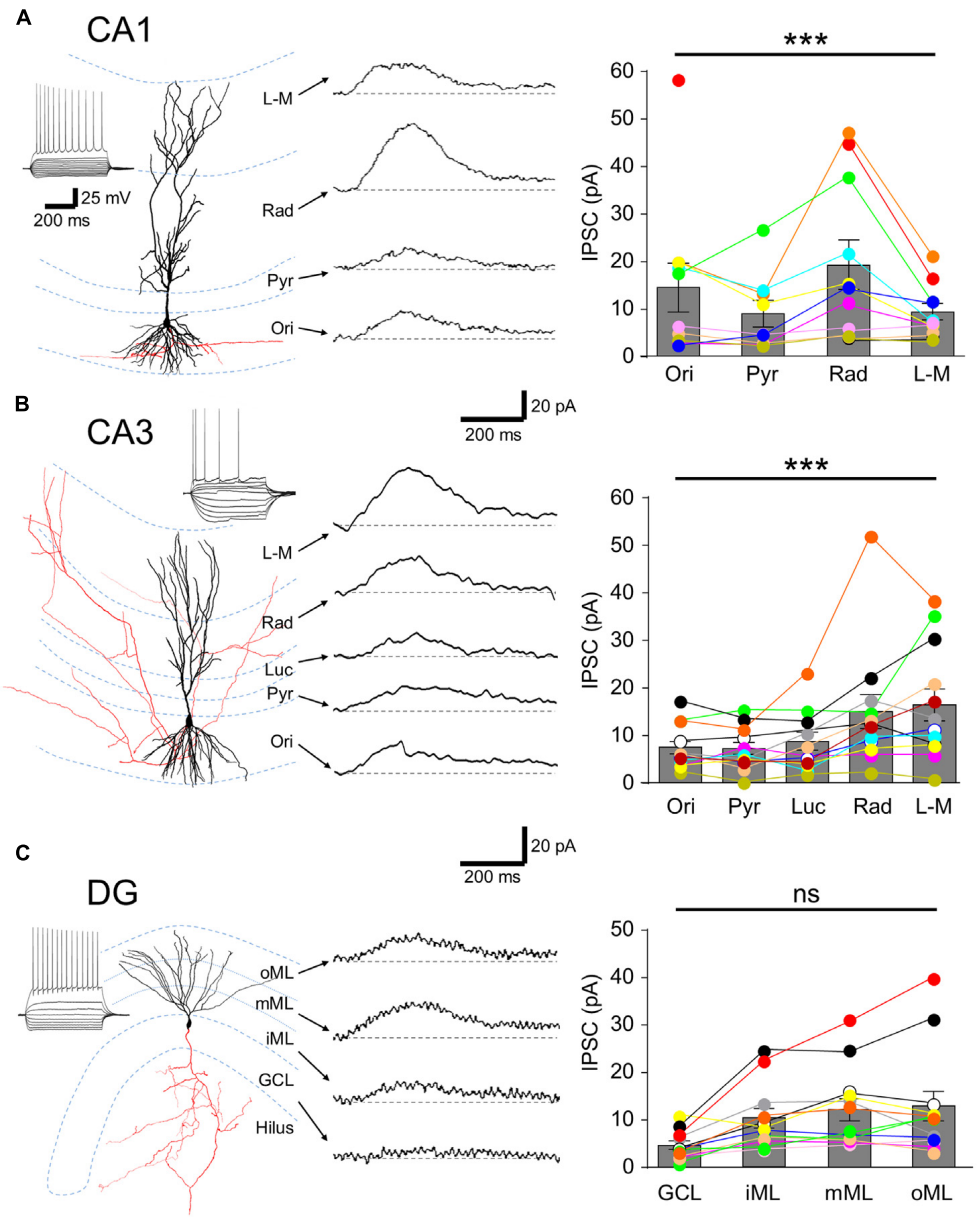
**Laminar distribution of GABA_**B**_R-mediated currents in hippocampal principal cells.** (**A**, left) Reconstruction of a CA1 PC. Soma and dendrites are shown in black and the axon in red. Inset, Voltage responses to a family of hyper- to depolarizing current pulses (–250 to +500 pA, 50 pA steps). A train of APs was elicited at the largest step. (**A**, middle) Representative pharmacologically isolated GABA_B_R-mediated uIPSCs in the different layers (L-M: *str. L-M*; Rad: *str. radiatum*; Pyr: *str. pyramidale*; Ori: *str. oriens*). Baseline is indicated by a gray dashed line, (**A**, right) Summary bar chart illustrates the mean uIPSC amplitudes for each layer (*n* = 11). (**B**, left) Reconstruction of a CA3 PC with the same layout as in **(A)** with the inset. (**B**, middle) GABA_B_R-mediated uIPSCs from a CA3 PC for the various layers (Luc: *str. lucidum*). (**B,** right) Summary bar chart of the mean uIPSC amplitudes for CA3 PCs (*n* = 12). (**C**, left) A reconstructed DGC, with intrinsic physiological response as inset. (**C**, middle) Representative GABA_B_R uIPSCs recorded from a DGC evoked in the different layers (GCL; iML, inner ML; mML, medial ML; oML, outer ML). (**C**, right) Summary bar chart of the mean uIPSC amplitudes for DGCs (*n* = 12). Bars for the mean values are overlain by data from individual experiments (color coded circles and lines). Statistics shown: ns = *P* > 0.05, ***P < 0.001; one-way ANOVA.

To assess the distribution GABA_B_Rs over the somatodendritic axis of hippocampal principal cells, we employed photolysis of caged GABA (Rubi-GABA, 20 μM) to map the laminar activation of the receptor-channel complexes (**Figure 2**). We first confirmed that uncaged Rubi-GABA mediated IPSCs (uIPSC) produced in the presence of NMDA and AMPA and GABA_A_ receptors blockers were mediated by GABA_B_Rs. To achieve this, we induced photolysis of Rubi-GABA in the entire visual field over the apical dendrites, which produced large amplitude slow uIPSCs in CA1 and CA3 PCs, as well as DGCs (52.6 ± 11.1, 71.7 ± 9.1, 29.1 ± 5.0 pA, respectively; data not shown). While these amplitudes were smaller than those during pharmacological activation, the differences in their magnitudes from the different cell types corresponded well to those for the baclofen induced currents (*P* = 0.005, one-way ANOVA). Furthermore, the decay-time constants of the full-field uIPSCs were similar to those elicited by a single extracellular stimulus (*P* > 0.05 all, Mann–Whitney test). Finally, in three CA1 PCs bath application of CGP reduced the uIPSC amplitude by 97% (data not shown), confirming that pharmacologically isolated slow uIPSCs were produced by the activation of postsynaptic GABA_B_Rs.

Spatially restricted uncaging of GABA was achieved by applying flashes of light (200 ms duration) to narrow strips (60 μm) in the different hippocampal layers perpendicular to the somatodendritic axis. In CA1 PCs (*n* = 11 cells) laminar activation of GABA_B_R produced uIPSCs with the largest amplitude in *str. radiatum* (19.2 ± 5.0 pA), followed by s*tr. oriens* with 14.4 ± 4.9 pA. uIPSCs elicited in the *str. L-M* and the perisomatic domain in the *str. pyramidale* had comparable amplitudes (9.3 ± 1.6 vs. 8.9 ± 2.7 pA). The observed differences between the layers were statistically significant (*P* = 0.0001, one-way ANOVA with Bonferonni multiple comparisons, **Figure 2A**). To confirm that laminar GABA_B_R-mediated responses were not due to extensive diffusion from the uncaging site, we photoreleased GABA in L6 of the cortex directly below the recorded CA1 PCs, which did not produce any discernible uIPSC (1.4 ± 0.2 pA, *P* < 0.05 compared to all other lamina).

As with CA1, uIPSCs in CA3 PCs (*n* = 13 cells) were large in *str. radiatum* and *L-M* but in these cells had similar mean amplitudes of 15.0 ± 3.4 and 16.4 ± 3.2 pA, respectively (*P* < 0.05 all, **Figure 2B**). uIPSCs in *str. oriens, lucidum* and *pyramidale*, were small with comparable amplitudes: 7.3 ± 1.2, 8.7 ± 1.8, and 7.2 ± 1.2 pA respectively (*P* > 0.05 all, **Figure 2B**, left); substantially lower than in *str. radiatum* and *L-M* (*P* = 0.0003, one-way ANOVA with Bonferroni multiple comparisons).

In contrast to PCs, lamina specific slow uIPSCs in DGCs (*n* = 13 cells) showed relatively constant amplitudes along the somatodendritic axis over different subregions of the ML (inner: 10.3 ± 2.0 pA, middle: 12.0 ± 2.2 pA and outer: 12.9 ± 3.0 pA; *P* > 0.05, **Figure 2C**), but had markedly lower mean amplitude of 4.6 ± 0.8 pA in the cell body layer (*P* = 0.0003, one-way ANOVA with Bonferroni multiple comparisons, **Figure 2C**).

In summary, GABA_B_R-mediated currents were observed in all somatodendritic domains of hippocampal principal cells but substantial differences were present in the laminar distribution of functional GABA_B_R-mediated currents, with high currents measured from the apical dendrites.

### LAMINAR DISTRIBUTION OF GABA_**B**_R-MEDIATED CURRENT AND CONDUCTANCE DENSITIES IN HIPPOCAMPAL PRINCIPAL CELLS

The Rubi-GABA uncaging-induced currents measured from the various layers showed large variability, which was at least partially due to differences in (1) the length and surface area of dendrites exposed to the light flash and (2) voltage-clamp errors and loss of currents at dendritic membranes. In order to estimate these factors, we have created 3-D reconstructions of a subset of neurons (five CA1 and five CA3 PCs, and four DGCs, **Table 2**), quantified dendritic length and surface area, then performed single cell simulations.

**Table 2:** Morphological and passive electrical properties of hippocampal principal cells used in the simulations.

Area	Cell code	Dendritic length (μm)	Dendritic surface (μm^2^)	Soma surface (μm^2^)	Input resistance (MΩ)	Membrane resistivity (Ωcm^2^)
**CA1**	20130613_02	10038	84706	1087	71.4	47200
	20130613_03	6539	44687	1246	78.1	22500
	20130617_02	9035	53584	1084	90.0	39050
	20130701_02	10763	67055	593	52.3	24000
	20131126_01	7675	66535	462	101.9	60700
**CA3**	20131106_02	8650	49925	1046	142.3	70900
	20131107_01	9191	36962	876	82.3	24600
	20131122_01	7679	41264	621	101.1	31300
	20131217_01	4677	27897	624	150.3	42550
	20131217_03	8454	46590	1304	134.9	59700
**DG**	20130613_01	2503	14514	318	66.0	6660
	20131112_01	3465	16520	238	95.1	9700
	20131117_02	3901	19827	403	130.8	17570
	20131121_03	2738	12861	402	106.1	8250

*Basic morphological and membrane properties of hippocampal principal cells as derived for single-cell simulations. The specific membrane capacitance (C_m_) was 1 μF/cm^*2*^, and the axial resistivity (Ri) 140 Ohm/cm for all cells, Membrane resistivity (R_m_) was assumed to be uniform for DGCs and non-uniform for PCs, with 50% lower values at the distal dendrites. The table refers to the proximal R_m_ value for the non-uniform models. For further detail see Methods*.

We first normalized the somatically measured current to the membrane surface area falling into the 60 μm-wid horizontal illumination windows. This normalization indicated that the current density for CA1 PCs was lowest in the perisomatic domain (0.91 ± 0.27 fA/μm^2^). The current density for the dendrites was moderately higher in the *str. oriens* (2.22 ± 1.15 fA/μm^2^) and *str. radiatum* (1.97 ± 0.94 fA/μm^2^), and it was the highest in the *str. L-M* (3.97 ± 0.80 fA/μm^2^), albeit with considerable cell-to-cell variability for all dendritic layers. In area CA3, the current densities showed a similar distribution with the highest values found at the apical dendrites in the *str. L-M* (10.3 ± 2.90 fA/μm^2^). In comparison to CA1 PCs, the normalized current values were approximately 2.5-times higher with the exception of the *str. oriens* which had the lowest value of all CA3 layers (1.15 ± 0.19 fA/μm^2^). In DG, the normalized currents were comparably low for the perisomatic domain as well as the proximal and medial dendrites in the inner and the middle ML (2.32–3.05 fA/μm^2^), but increased markedly for the distal dendrites in the outer ML (7.92 ± 4.71 fA/μm^2^).

**FIGURE 3:**
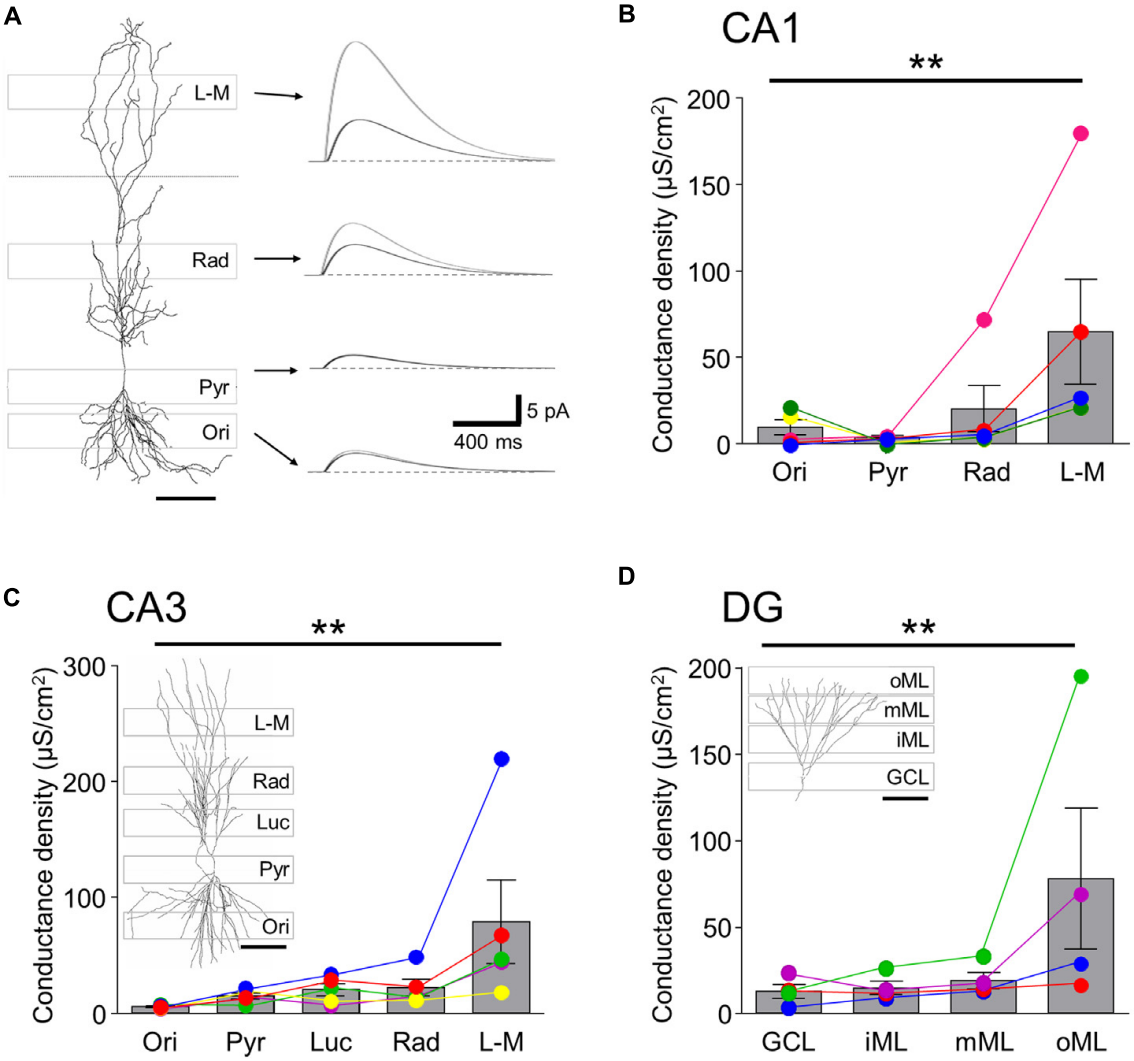
**Single-cell simulation of GABA_**B**_R mediated currents and conductance densities in 3-D reconstructed hippocampal neurons.**(**A**, left) 3-D reconstruction of a CA1 PC shown in conjunction with the laminar stripes in which Rubi-GABA was released (gray boxes overlain). (**A,** right) Simulated GABA_B_R-mediated ISPC pairs at the somatic recording site (black traces) and the illuminated perisomatic or dendritic membranes (gray traces) corresponding to the layer specific photostimulation windows (indicated by the arrows). The dendritic conductances were set so that the somatic IPSC amplitudes match the experimentally observed light induced current amplitudes. Note the difference in the amplitudes of the dendritic and somatic currents. **(B)** Summary bar chart showing the GABA_B_R-mediated conductance densities for the illumination windows in the different layers of the CA1 area derived in the simulations. Average data is overlain by data from individual experiments (colored circles and lines). Note that the conductance densities were consistently largest in *str. L-M*. **(C,D)** Summary bar charts showing the conductance densities for membrane surfaces in the illumination windows in the different layers for CA3 PCs and DGCs, respectively; same format as in **(B)**. Representative cell reconstructions with the illumination windows superimposed are shown as insets. Scale bars: 100 μm. Statistics shown: **P < 0.01, from Friedman’s tests.

In order to estimate the local GABA_B_R-mediated conductance densities in the different somatodendritic compartments, we next ran single-cell simulations using the reconstructed principal cell models assuming passive membrane properties (**Table 2** and **Figure 3**). As expected, the conductance density for the perisomatic domain of CA1 showed the lowest mean value (G_Mem_ = 3.7 ± 1.0 μS/cm^2^, **Figures 3A,B**) and the lowest cell-to-cell variability. It was very close to the estimated value (G_est_ = 3.0 ± 0.8 μS/cm^2^) obtained from the normalized current density and the driving force, confirming that there was very little loss when measuring the induced GABA_B_R-mediated slow currents in this compartment. For the dendrites in the *str. oriens* conductance densities (G_Mem_ = 9.5 ± 4.9 μS/cm^2^, **Figure 3B**) were higher both in absolute value and in comparison to the estimated density from the current densities. Nevertheless, the ratio of simulated and estimated conductance densities (G_Mem_/G_est_ = 78.3%) indicated only a moderate attenuation along the basal dendrites. By contrast, in *str. radiatum* and *str. L-M*, the majority of the dendritic segments were dominated by small caliber oblique and tuft branches, the density values were markedly higher (20.3 ± 15.0 and 64.7 ± 33.8 μS/cm^2^, respectively) and the attenuation stronger (G_Mem_/G_est_ = 48.6 and 31.7%, respectively), which was highly significantly different, as shown by a Friedman test (*P* = 0.006), and also confirmed that *str. L-M* had a higher conductance density than basal dendrites in the *str. oriens* (*P* < 0.05).

In the CA3 region the conductance density was lowest in the *str. oriens* (5.2 ± 0.7 μS/cm^2^, **Figure 3C**), comparable to that of CA1 in terms of both its amplitude and the degree of attenuation. Interestingly, the densities for the perisomatic domain and proximal and middistal dendrites in the *str. lucidum* and *radiatum* had similar conductance densities of between 14.5 and 21.8 μS/cm^2^ (*P* > 0.05 all, Friedman’s test). The conductance density in CA3 PCs was highest in *str. L-M*, with a value (78.9 ± 40.3 μS/cm^2^, *P* < 0.05) which was slightly larger, but not significantly so to that for CA1 PCs (*P* = 0.67, Mann–Whitney test). However, the attenuation was less pronounced (G_Mem_/G_est_ = 54.8%) from the distal apical dendrites of CA3 PCs, explaining the larger observed somatic currents.

Finally, in DGCs the conductance density increased incrementally from the GCL toward the middle ML from 12.1 to 18.6 μS/cm^2^, whereas it was markedly higher for the distal apical dendrites in the outer ML (77.3 ± 47.0 μS/cm^2^; *P* < 0.05 inner vs. outer ML, **Figure 3D**). Attenuation in the dendrites of GCs was less pronounced than in apical dendrites of CA1 PCs: the G_Mem_/G_est_ was 82.0% at the perisomatic domain, reflecting minimal attenuation, decreasing consistently toward the distal dendrites of the outer ML (34.9%).

In summary, the morphometric analysis and the single-cell simulations further confirmed that GABA_B_R-mediated currents were observed in all somatodendritic compartments of hippocampal principal cells and revealed that while on the perisomatic domain and the basal dendrites of PCs conductance densities are low, they are high on the distal apical dendrites in the *str L-M* and in the oML.

### DISTRIBUTION OF GABA_**B1**_ AND Kir3.2 SUBUNITS IS DIFFERENT BETWEEN HIPPOCAMPAL SUBFIELDS AND LAMINA AS SHOWN BY SDS-FRL

As previous work (Kulik et al., 2003, 2006) has shown light microscopic differences in laminar expression of GABA_B_ and Kir3 subunits, we asked whether the distribution of GABA_B_R-mediated currents is concordant with GABA_B_R and Kir3 channel expression at the plasma membrane along the somatodendritic axis of hippocampal principal cells. We therefore performed highly sensitive SDS-FRL labeling for these signaling molecules (Hagiwara et al., 2005; Kulik et al., 2006) to allow us to accurately calculate the membrane surface densities of these proteins. Immunogold labeling for GABA_B1_ and Kir3.2 subunits was achieved from replicas containing all dendritic and somatic layers for each region, so that the laminar distribution could be compared under identical conditions for each hippocampal region. Consistent with previous reports from pre-embedding immunogold labeling (Kulik et al., 2003, 2006; Booker et al., 2013) GABA_B1_ and Kir3.2 subunits were found at high densities on the postsynaptic membrane of the entire somatodendritic axis of principal cells.

**FIGURE 4:**
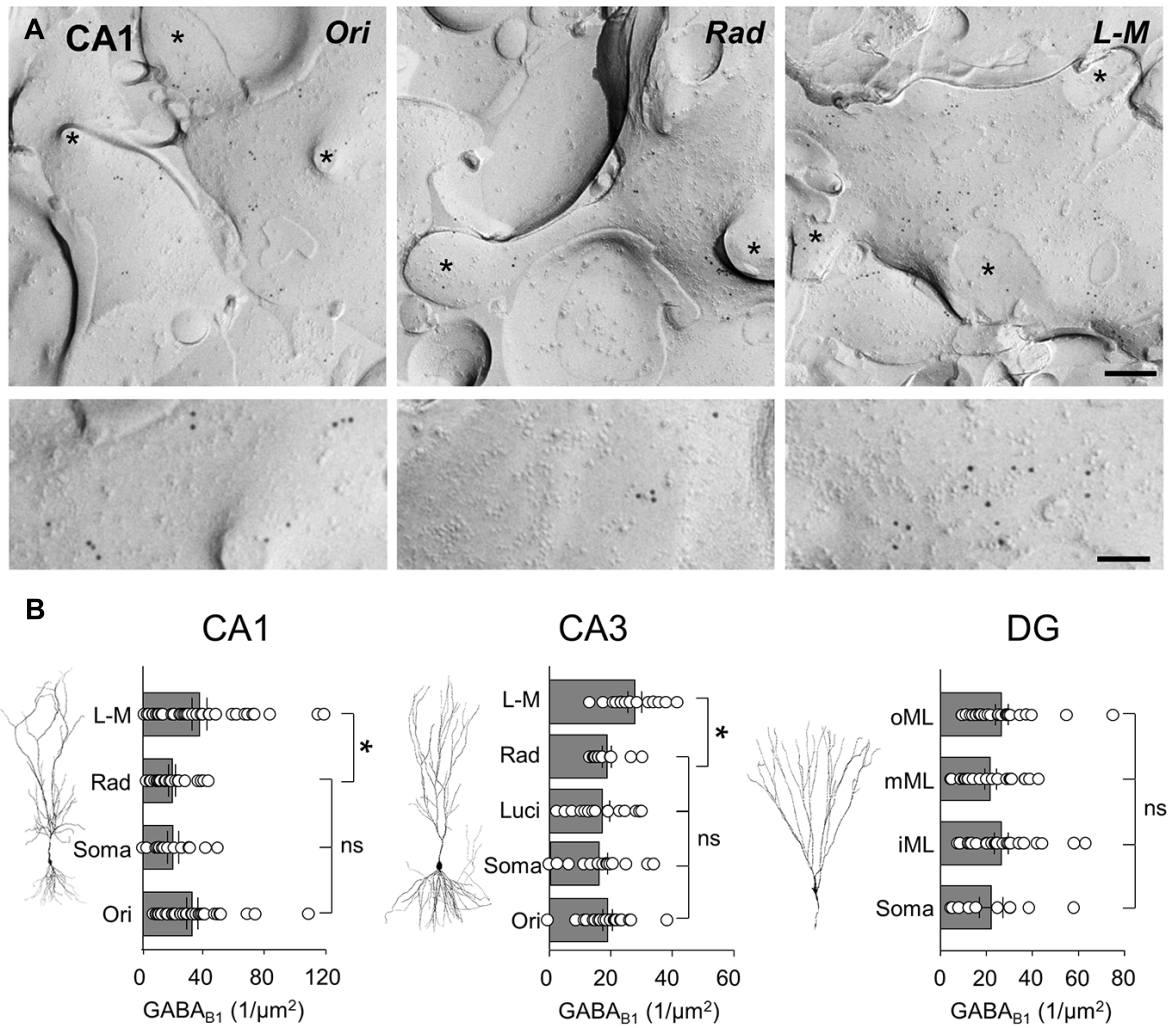
**GABA_**B1**_ receptor subunits are differentially expressed along the somatodendritic axis of hippocampal principal cells. (A)** Representative SDS-FRL electron micrographs of spiny dendrites from str. oriens (Ori), str. radiatum (Rad), and stratum L - M (L - M) of the CA1. The putative CA1 PC dendrites show immunogold labeling for GABA_B1_ receptor subunits (10 nm gold particles). Spines and fractured spine necks are indicated with asterisks (^∗^). Scale bar: 200 nm (top row), 100 nm (bottom row). **(B)** Summary bar charts of the GABA_B1_ subunit surface density from somatodendritic compartments in different layers of CA1 (left), CA3 (middle), and DG (right) are shown alongside representative images of principal cells. Mean surface densities are overlain by density measurements of individual P-face structures. Statistics shown: ns = *P* > 0.05, *P < 0.05, one-way ANOVA.

In CA1, spiny dendritic and somatic profiles were collected from all dendritic layers and the *str. pyramidale*, respectively (**Figure 4A**). Quantification of immunogold particles for GABA_B1_ subunit revealed that the labeling intensity varied substantially between the different layers (*P* = 0.0053, one-way ANOVA, **Figure 4B**, left). Immunogold particle density was highest in *str. L-M* and *oriens* which had a similar density of labeling (36.8 ± 4.9 and 32.1 ± 3.6 particles/μm^2^, respectively; *P* > 0.05 one-way ANOVA with Bonferroni multiple comparisons). Dendrites in *str. radiatum* and somatic membranes of CA1 PCs showed lower labeling with similar levels (19.0 ± 2.3 and 19.6 ± 3.7 particles/μm^2^, respectively; *P* = 0.9, one-way ANOVA with Bonferroni multiple comparisons) which was approximately 50% of those found on the basal and distal apical dendrites (**Figure 4B**, left).

In CA3 PCs, immunogold particle density for the GABA_B1_ subunits was highest on dendrites in the *str. L-M* (27.9 ± 2.3 particles/μm^2^, **Figure 4B**, middle), similar to the CA1. In the other CA3 layers, however, putative PC spiny dendrites as well as somata showed a 30–40% lower, but broadly similar labeling intensities (str. oriens: 19.0 ± 1.6 particles/μm^2^, CA3 PC somata: 15.9 ± 2.6 particles/μm^2^, *str. lucidum*: 17.4 ± 2.4 particles/μm^2^, *str. radiatum*: 18.7 ± 1.5 particles/μm^2^, **Figure 4B**, middle, *P* = 0.0019, one-way ANOVA comparison of all layers), Finally, in the DG the membrane surface densities for the GABA_B1_ subunits were largely constant across the layers (*P* = 0.59, one-way ANOVA with multiple comparisons, **Figure 4B**, right), with surface densities of 22.1 ± 5.1 particles/μm^2^ on somata in the GCL, 26.6 ± 3.1 particles/μm^2^ on dendrites in the inner ML, 21.9 ± 2.5 particles/μm^2^ in the middle ML, and finally 26.6 ± 2.8 particles/μm^2^ in the outer ML.

**FIGURE 5:**
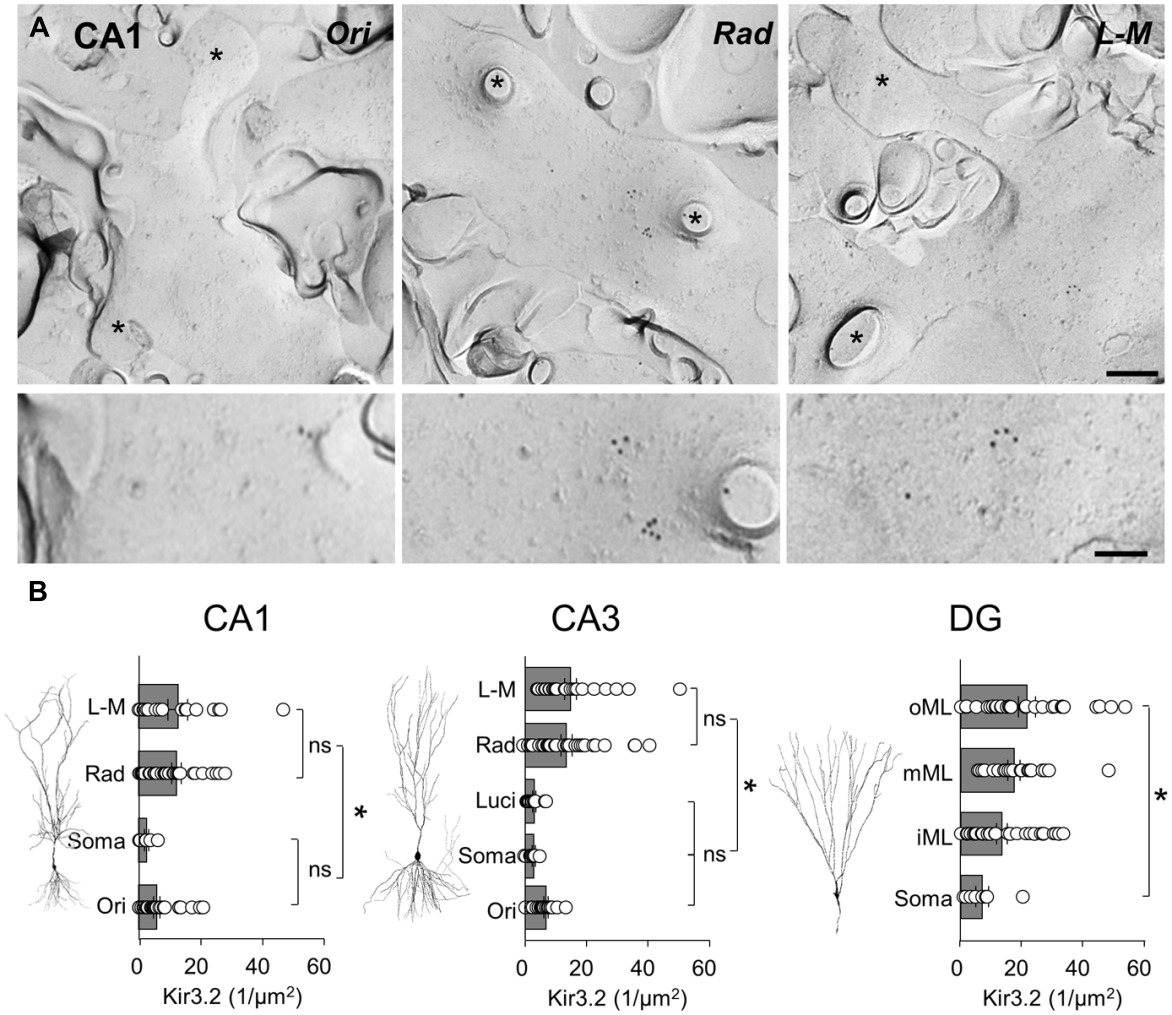
**The Kir3.2 channel subunit is differentially expressed along the somatodendritic axis of hippocampal principal cells. (A)** Representative electron micrographs of spiny dendrites from str. oriens (Ori), radiatum (Rad) and L - M (L - M) of the CA1. Immunogold labeling for the Kir3.2 channel subunit seen as 10 nm gold particles. Spines and fractured spine necks are indicated with asterisks (^∗^). Scale bar: 200 nm (top row), 100 nm (bottom row). **(B)** Summary bar charts of the surface density for the Kir3.2 subunit from the different layers of the CA1 (left), CA3 (middle), and DG (right) are shown alongside representative images of principal cells. Mean surface densities are overlain by measurements of individual density values for examined structures. Statistics shown: ns = *P* > 0.05, *P < 0.05, one-way ANOVA.

Localization of the constitutive Kir3 effector channel subunit, Kir3.2, in spiny dendrites and somata of CA1 showed a generally lower, but largely overlapping pattern of labeling, with some notable differences when compared to that of GABA_B1_. Dendrites in the CA1, s*tr. radiatum* and *L-M* showed a high and relatively constant membrane surface labeling (12.1 ± 1.5 and 12.8 ± 3.2 particles/μm^2^, respectively, *P* > 0.05; **Figure 5A,B**, left). Immunogold particle density was ∼50% lower on dendrites in *str. oriens* (5.9 ± 1.1 particles/μm^2^) and ∼80% lower on the somatic membrane in the *str. pyramidale* (2.6 ± 0.8 particles/μm^2^), which were significantly different from *str. radiatum* and *L-M*, (*P* = 0.0013, one-way ANOVA with Bonferonni multiple comparisons; **Figure 5A,B**, left). A similar pattern was observed in CA3 (*P* < 0.0001, one-way ANOVA, **Figure 5B**, middle), where Kir3.2 labeling was high in the *str. radiatum* and *L-M* (13.4 ± 1.8 and 14.7 ± 1.9 particles/μm^2^, respectively), 50% lower in the *str. oriens* (6.8 ± 0.6 particles/μm^2^) and even lower in dendrites in *str. lucidum* and on somata in the *str. pyramidale* (3.0 ± 0.5 and 2.8 ± 0.5 particles/μm^2^, respectively). Finally, in the DG, the labeling for Kir3.2 in the somatodendritic domain of DGCs showed a gradual increase from the soma to the distal dendrites (*P* = 0.006, one-way ANOVA, **Figure 5B**, right), with mean Kir3.2 densities of 7.7 ± 2.3, 14.8 ± 1.9, 19.2 ± 2.2, and 23.8 ± 3.0 particles/μm^2^ on somata in the GCL, and dendrites in the inner, middle and outer ML, respectively.

In summary, SDS-FRL labeling for GABA_B1_ and Kir3.2 subunits showed clear lamina- and region-specific differences. GABA_B1_ displayed higher expression with moderate differences along the somatodendritic axis of principal cells. In contrast, labeling for Kir3.2 had a lower overall level, but the labeling density was consistently higher on distal apical dendrites than on basal, proximal apical dendrites or somata.

## DISCUSSION

**FIGURE 6:**
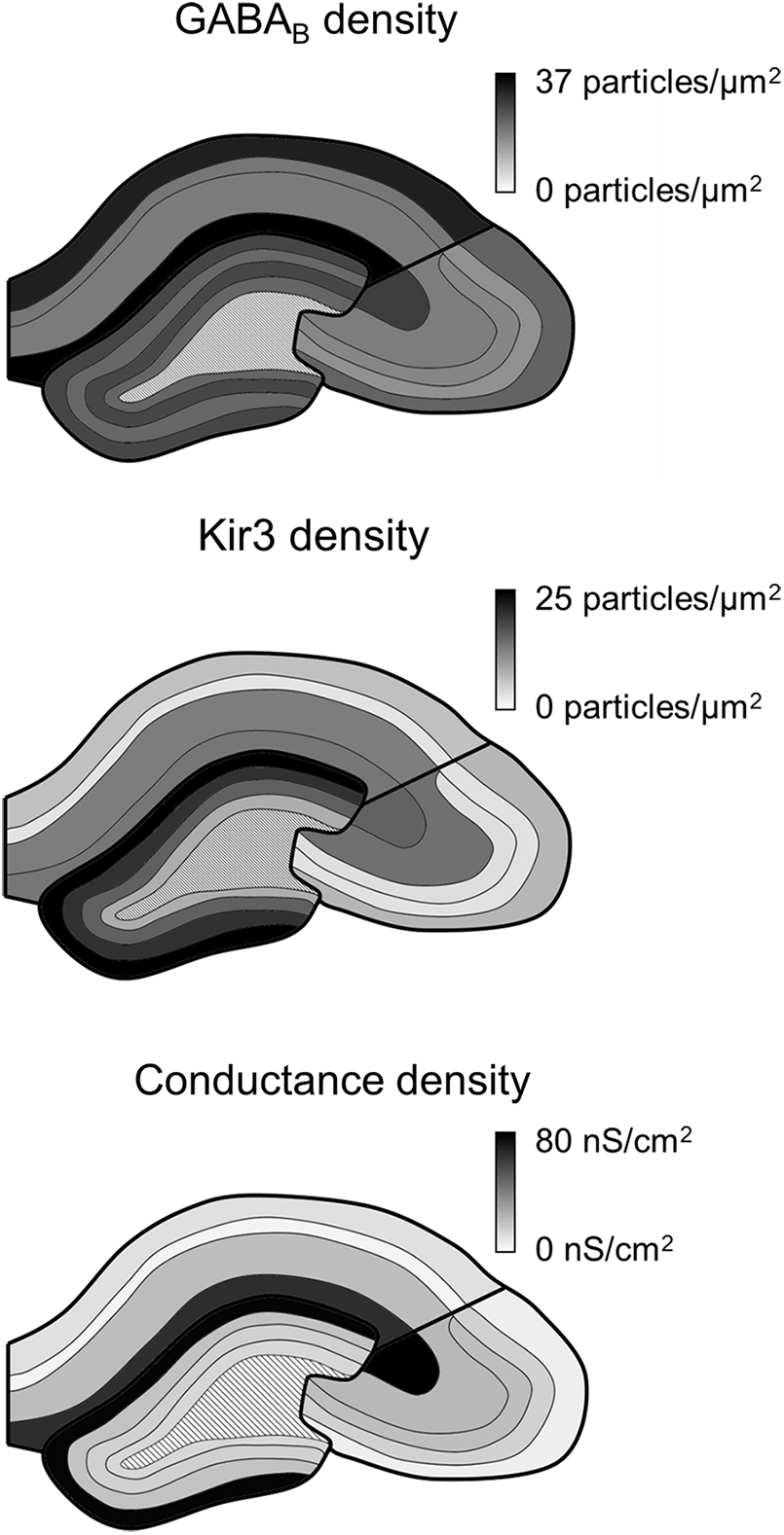
**Summary of GABA_**B**_ receptor and Kir3 effector channel and conductance density distributions in the hippocampus.** Schematic overviews of the hippocampus show the density of immunogold labeling for GABA_B_ receptor (top) and Kir3 channels (middle) as well as for conductance densities derived in the simulations (bottom). Boundaries of the hippocampal areas CA1, CA3, and DG as well as their layers are delineated in black; dark to light tones correspond to high to low densities (see scale bars to the right).

Our study confirms that the GABA_B_R-Kir3 channel signaling cascade is present along the entire somatodendritic axis of hippocampal principal cells. However, the receptor and effector channel, as well as the resulting conductances are not uniformly distributed, but show region- and layer-specific differences (**Figure 6**). Receptor and channel distributions are overlapping, but show divergence, whereby the conductance densities and somatic current amplitudes correlate better with Kir3 densities, suggesting that GABA_B_R-mediated currents are primarily determined by an effector-bottlenecking effect.

### REGIONAL DIFFERENCES IN GABA_**B**_R EXPRESSION AND CURRENTS

The observed GABA_B_R-mediated currents varied between hippocampal regions with the highest currents in CA3 PCs. This difference is consistent with previous light microscopic studies, which showed higher intensity GABA_B_R labeling in the CA3 neuropil (Fritschy et al., 1999; Sloviter et al., 1999; Kulik et al., 2003). However, high-resolution analysis of GABA_B1_ subunits on dendritic membranes revealed a different relationship whereby CA1 > CA3 > DG. This discrepancy between the whole-cell currents and the immunogold densities in SDS-FRL might be explained by a larger somatodendritic surface of CA3 PCs (Cannon et al., 1999; note however, that in our sample such a trend wasn’t present), divergent GABA_B1_ splice-variant localization, or variation in immunogold labeling between replicas. Furthermore, amplitudes of GABA_B_R-mediated currents are likely to be limited by Kir3 channel densities, which were comparably low in the two cell types. In good agreement, the low I_WC_ measured in DGCs was consistent with the smaller dendritic arbor and low receptor and channel expression. However, this result is in conflict with the proposition that DGCs receive stronger inhibitory input (Gottlieb and Cowan, 1972; Bekenstein and Lothman, 1991) although GABA_A_- vs. GABA_B_-mediated mechanisms may show divergence.

### LAMINA SPECIFIC DIFFERENCES IN GABA_**B**_R AND Kir3 CHANNEL DENSITY

Our data further show that GABA_B_Rs, as well as effector Kir3 channels are differentially localized to somatodendritic compartments of hippocampal principal cells (See **Figure 6**). This is in line with results of previous light microscopic investigations (Fritschy et al., 1999; Sloviter et al., 1999; Kulik et al., 2003). SDS-FRL method enables high-resolution subcellular localization and label a high proportion (>70%) of surface-localized proteins (Tanaka et al., 2005). Therefore, the membrane densities of both GABA_B1_ and Kir3.2 subunits detected in the electron microscope are a better reflection of their subcellular distributions than light microscopic data. In fact the currents measured for the various layers correlated well with the densities of Kir3.2 in the electron microscope. This result further suggests that coupling efficiency between the receptor and the channel might be spatially uniform but the currents are largely defined by the availability of Kir3.2-containing channels.

Previous work has indicated that GABA_B_R-mediated IPSPs in CA1 are uniform across dendritic layers in terms of amplitudes and kinetics (Pham et al., 1998). Our physiological examination of GABA_B_R-mediated currents evoked by lamina-specific photolysis of caged-GABA now reveals differential expression of the currents in all regions of the rat hippocampus with highest magnitudes on the distal apical dendrites. However, the somatically recorded responses do not account for the electrotonic properties of the neurons (Spruston et al., 1993; Szilágyi and De Schutter, 2004). Indeed, simulations using morphologically detailed single-cell models demonstrate that the somatic voltage-clamp recordings substantially underestimate conductances in distal dendritic compartments.

As our single cell model included several assumptions, we need to exert caution with the interpretation of the quantitative results (Szilágyi and De Schutter, 2004). Nevertheless, the highest conductance densities obtained for the distal apical dendrites (65–80 μS cm^-2^) suggest that during flash-induced activation 0.02–0.1 channels are open on a 1-μm^2^ membrane patch (assuming a single-channel conductance between 5 and 31 pS, Takigawa and Alzheimer, 1999; Chen and Johnston, 2005). This number is markedly lower than the density of immunogold particles for both the receptor and effector channel in the same compartments (10–40/μm^2^) suggesting that less than 1% of the channels are open upon receptor activation. Even if we consider the low open probability of the activated channels (0.119, Chen and Johnston, 2005), the coupling of receptor and channel appears to be inefficient in both interneurons (Booker et al., 2013) and principal cells.

A finding of major interest is the high GABA_B_R/Kir3 conductance density on the distal apical dendrites of hippocampal principal cells. This is at odds with results from layer 5 of the somatosensory cortex (Breton and Stuart, 2012), which suggested that GABA_B_R effects are mediated by inhibition of voltage-sensitive calcium channels, but not Kir3 channels in distal dendrites. Our data show that this hypothesis cannot be extended to the hippocampus, as the highest expression of Kir3 channels, and the largest conductances were detected in the distal apical dendrites. In fact, this canonical postsynaptic signaling cascade was present along the entire somatodendritic axis of hippocampal PCs. However, this finding does not preclude that receptors interact with other effectors. On the contrary, the apparently inefficient coupling of receptor and Kir3 channels could be explained by divergent signaling cascades. Furthermore, it is plausible that activation of Kir3 channels and inhibition of L-type calcium channels act synergistically to control dendritic electrogenesis and plasticity in distal apical dendrites (Palmer et al., 2012; Larkum, 2013; Pérez-Garci et al., 2013).

The current results raise questions about the high membrane expression of GABA_B1_ in the absence of high Kir3 channel expression, as seen on basal dendrites in *str. oriens* or the perisomatic domain of CA1 PCs. What the function of this enrichment in GABA_B_R compared to the effector is, remains unclear. The simplest explanation could be that a larger number of GABA_B_Rs are needed to ensure high fidelity inhibition. Indeed, on PC dendrites in *str. oriens* only ∼3% of synaptic contacts are inhibitory (Megías et al., 2001), suggesting a low availability of GABA in the extrasynaptic space. Alternatively, GABA_B_Rs may couple to other effector systems, such as calcium channels (Carter and Mynlieff, 2004; Chalifoux and Carter, 2011; Breton and Stuart, 2012). Furthermore, GABA_B_Rs have been shown to inhibit NMDA receptor mediated calcium influx (Chalifoux and Carter, 2010) and to enhance activity of group 1 mGluRs (Hirono et al., 2001) and GABA_A_R (Connelly et al., 2013; Tao et al., 2013); all perhaps explaining the excess of GABA_B_Rs in *str. oriens*. Furthermore, GABA_B_Rs have been shown to stimulate the transcription factors CREB2 and ATFx (Nehring et al., 2000; White et al., 2000); this could explain the presence of surface localized somatic GABA_B1_, but not Kir3.2 in all hippocampal principal cells.

### FUNCTIONAL IMPLICATIONS FOR LAMINAR SPECIFIC DIFFERENCES IN FUNCTIONAL GABA_**B**_R-MEDIATED CURRENTS

We have observed GABA_B_R, Kir3 channels and their functional currents in all somatodendritic regions of hippocampal principal cells. This will have substantial repercussions for the activity of the hippocampal microcircuit, by providing slow inhibition which shapes the excitability of dendrites and modulates synaptic plasticity (Kohl and Paulsen, 2010). The presence of large functional currents on distal apical dendrites, which receive input from the perforant path, is particularly pertinent, as this pathway lacks strong presynaptic GABA_B_R-mediated inhibition (Lanthorn and Cotman, 1981). Thus, at distal dendritic locations, GABA_B_R-mediated inhibition mostly arises from postsynaptic receptors. By comparison, the Schaffer collateral pathways are strongly inhibited by GABA_B_R activation (Lanthorn and Cotman, 1981; Ault and Nadler, 1982). Therefore, excitatory transmission in these pathways is subjected to strong pre- and postsynaptic GABA_B_R-mediated inhibition in *str. radiatum*, whereas in *str. oriens* presynaptic effects will dominate. In view of the differential affinity of pre- and postsynaptic GABA_B_Rs to GABA (Dugladze et al., 2013), differences along the somatodendritic axis have profound effects on synaptic integration and activation of principal cells as a function of GABA levels. Furthermore, synaptic plasticity is likely to be modulated differentially at these inputs by GABA_B_Rs, as presynaptic inhibition, postsynaptic membrane hyperpolarization and inhibition of NMDA receptors have negative impact on Hebbian plasticity (Magee and Johnston, 1997). This issue, however, is confounded by disinhibitory effects, produced via GABA_B_R activation in inhibitory interneurons (Buhl et al., 1996; Mott et al., 1999; Booker et al., 2013), which enhances LTP (Mott et al., 1989, 1990; Davies et al., 1991). In good agreement with this proposition, LTP is larger in CA1 *str. oriens* than in str. *radiatum* (Haley et al., 1996; Bradshaw et al., 2003).

Finally, the presence of GABA_B_R-mediated currents over the entire somatodendritic axis of hippocampal principal cells suggests that inhibitory interneurons, with axon projecting to any lamina, can contribute to GABA_B_R IPSCs through volume transmission (Isaacson and Nicoll, 1993; Scanziani, 2000; Booker et al., 2013). This conclusion is in conflict with the hypothesis that certain types of interneuron, neurogliaform and ivy cells, are the source of GABA_B_R-mediated inhibition (Williams and Lacaille, 1992; Price et al., 2005, 2008; Olah et al., 2009). Therefore it seems likely, that although interneurons with dense, focal axon among dendrites are capable of producing large GABA_B_R currents, all interneurons could contribute to these responses.

In conclusion, we have shown that the GABA_B_R-Kir3 signaling cascade is present along the entire somatodendritic axis of hippocampal principal cells, but show region- and lamina-specific distributions. These differences will impact dendritic integration, synaptic plasticity and the emerging principal cell activity for inputs from the various afferent pathways during heightened GABAergic activity.

## Conflict of Interest Statement

The authors declare that the research was conducted in the absence of any commercial or financial relationships that could be construed as a potential conflict of interest.

## Footnotes

1 http://www.stimfit.org

2 http://fiji.org

## References

[B1] Ault B. Nadler J. V. (1982). Baclofen selectively inhibits transmission at synapses made by axons of CA3 pyramidal cells in the hippocampal slice. *J. Pharmacol. Exp. Ther.* 223 291–297. 6290635

[B2] Baker J. Perez-Rosello T. Migliore M. Barrionuevo G. Ascoli G. (2011). A computer model of unitary responses from associational/commissural and perforant path synapses in hippocampal CA3 pyramidal cells. *J. Comput. Neurosci.* 31 137–158 10.1007/s10827-010-0304-x 21191641 PMC4164295

[B3] Bekenstein J. W. Lothman E. W. (1991). A comparison of the ontogeny of excitatory and inhibitory neurotransmission in the CA1 region and dentate gyrus of the rat hippocampal formation. *Dev. Brain Res.* 63 237–243 10.1016/0165-3806(91)90083-U 1665107

[B4] Bettler B. Kaupmann K. Mosbacher J. Gassmann M. (2004). Molecular structure and physiological functions of GABA_B_ receptors. *Physiol. Rev.* 84 835–867 10.1152/physrev.00036.2003 15269338

[B5] Booker S. A. Gross A. Althof D. Shigemoto R. Bettler B. Frotscher M. (2013). Differential GABA_B_-receptor-mediated effects in perisomatic- and dendrite-targeting parvalbumin interneurons. *J. Neurosci.* 33 7961–7974 10.1523/JNEUROSCI.1186-12.2013 23637187 PMC3814621

[B6] Bradshaw K. D. Emptage N. J. Bliss T. V. P. (2003). A role for dendritic protein synthesis in hippocampal late LTP. *Eur. J. Neurosci.* 18 3150–3152 10.1111/j.1460-9568.2003.03054.x 14656312

[B7] Breton J.-D. Stuart G. J. (2012). Somatic and dendritic GABA_B_ receptors regulate neuronal excitability via different mechanisms. *J. Neurophysiol.* 108 2810–2818 10.1152/jn.00524.2012 22956789

[B8] Buhl E. H. Szilágyi T. Halasy K. Somogyi P. (1996). Physiological properties of anatomically identified basket and bistratified cells in the CA1 area of the rat hippocampus in vitro. *Hippocampus* 6 294–305 10.1002/(SICI)1098-1063(996)6:3<294::AID-HIPO7>3.0.CO;2-N 8841828

[B9] Cannon R. C. Wheal H. V. Turner D. A. (1999). Dendrites of classes of hippocampal neurons differ in structural complexity and branching patterns. *J. Comp. Neurol.* 413 619–633 10.1002/(SICI)1096-9861(19991101)413:4<619::AID-CNE10>3.0.CO;2-B 10495447

[B10] Carnevale N. T. Hines M. L. (2009). *The NEURON Book.* Cambridge: Cambridge University Press.

[B11] Carter T. J. Mynlieff M. (2004). Gamma-aminobutyric acid type B receptors facilitate L-type and attenuate N-type Ca2+ currents in isolated hippocampal neurons. *J. Neurosci. Res.* 76 323–333 10.1002/jnr.20085 15079861

[B12] Chalifoux J. R. Carter A. G. (2010). GABA_B_ receptors modulate NMDA receptor calcium signals in dendritic spines. *Neuron* 66 101–113 10.1016/j.neuron.2010.03.012 20399732 PMC2861500

[B13] Chalifoux J. R. Carter A. G. (2011). GABA_B_ receptor modulation of voltage-sensitive calcium channels in spines and dendrites. *J. Neurosci.* 31 4221–4232 10.1523/JNEUROSCI.4561-10.2011 21411663 PMC3061967

[B14] Chen X. Johnston D. (2005). Constitutively active G-protein-gated inwardly rectifying K+ channels in dendrites of hippocampal CA1 pyramidal neurons. *J. Neurosci.* 25 3787–3792 10.1523/JNEUROSCI.5312-04.2005 15829630 PMC6724929

[B15] Connelly W. M. Fyson S. J. Errington A. C. McCafferty C. P. Cope D. W. Di Giovanni G. (2013). GABA_B_ receptors regulate extrasynaptic GABAA receptors. *J. Neurosci.* 33 3780–3785 10.1523/JNEUROSCI.4989-12.2013 23447590 PMC3601669

[B16] Davies C. H. Starkey S. J. Pozza M. F. Collingridge G. L. (1991). GABA_B_ autoreceptors regulate the induction of LTP. *Nature* 349 609–611 10.1038/349609a0 1847993

[B17] Drake C. T. Bausch S. B. Milner T. A. Chavkin C. (1997). GIRK1 immunoreactivity is present predominantly in dendrites, dendritic spines, and somata in the CA1 region of the hippocampus. *Proc. Natl. Acad. Sci. U.S.A.* 94 1007–1012 10.1073/pnas.94.3.1007 9023373 PMC19630

[B18] Dugladze T. Maziashvili N. Börgers C. Gurgenidze S. Häussler U. Winkelmann A. (2013). GABA_B_ autoreceptor-mediated cell type-specific reduction of inhibition in epileptic mice. *Proc. Natl. Acad. Sci. U.S.A.* 110 15073–15078 10.1073/pnas.1313505110 23980149 PMC3773756

[B19] Dutar P. Nicoll R. A. (1988). Pre- and postsynaptic GABA_B_ receptors in the hippocampus have different pharmacological properties. *Neuron* 1 585–591 10.1016/0896-6273(88)90108-0 2856099

[B20] Fritschy J.-M. Meskenaite V. Weinmann O. Honer M. Benke D. Mohler H. (1999). GABA_B_-receptor splice variants GB1a and GB1b in rat brain: developmental regulation, cellular distribution and extrasynaptic localization. *Eur. J. Neurosci.* 11 761–768 10.1046/j.1460-9568.1999.00481.x 10103070

[B21] Golding N. L. Mickus T. J. Katz Y. Kath W. L. Spruston N. (2005). Factors mediating powerful voltage attenuation along CA1 pyramidal neuron dendrites. *J. Physiol.* 568 69–82 10.1113/jphysiol.2005.086793 16002454 PMC1474764

[B22] Gottlieb D. Cowan W. M. (1972). On the distribution of axonal terminals containing spheroidal and flattened synaptic vesicles in the hippocampus and dentate gyrus of the rat and cat. *Z. Zellforsch. Mikrosk. Anat.* 129 413–429 10.1007/BF00307297 5042776

[B23] Guzman S. J. Schlgl A. Schmidt-Hieber C. (2014). Stimfit: quantifying electrophysiological data with Python. *Front. Neuroinform.* 8:16 10.3389/fninf.2014.00016 PMC3931263 24600389

[B24] Hagiwara A. Fukazawa Y. Deguchi-Tawarada M. Ohtsuka T. Shigemoto R. (2005). Differential distribution of release-related proteins in the hippocampal CA3 area as revealed by freeze-fracture replica labeling. *J. Comp. Neurol.* 489 195–216 10.1002/cne.20633 15983999

[B25] Hájos N. Ellender T. J. Zemankovics R. Mann E. O. Exley R. Cragg S. J. (2009). Maintaining network activity in submerged hippocampal slices: importance of oxygen supply. *Eur. J. Neurosci.* 29 319–327 10.1111/j.1460-9568.2008.06577.x 19200237 PMC2695157

[B26] Haley J. E. Schaible E. Pavlidis P. Murdock A. Madison D. V. (1996). Basal and apical synapses of CA1 pyramidal cells employ different LTP induction mechanisms. *Learn. Mem.* 3 289–295 10.1101/lm.3.4.289 10456098

[B27] Hines M. L. Carnevale N. T. (1997). The NEURON simulation environment. *Neural. Comput.* 9 1179–1209 10.1162/neco.1997.9.6.1179 9248061

[B28] Hirono M. Yoshioka T. Konishi S. (2001). GABA_B_ receptor activation enhances mGluR-mediated responses at cerebellar excitatory synapses. *Nat. Neurosci.* 4 1207–1216 10.1038/nn764 11704764

[B29] Isaacson J. S. Nicoll R. A. (1993). The uptake inhibitor L-*trans*-PDC enhances responses to glutamate but fails to alter the kinetics of excitatory synaptic currents in the hippocampus. *J. Neurophysiol.* 70 2187–2191. 7905032 10.1152/jn.1993.70.5.2187

[B30] Kohl M. M. Paulsen O. (2010). The roles of GABA_B_ receptors in cortical network activity. *Adv. pharmacol.* 58 205–229 10.1016/S1054-3589(10)58009-8 20655484

[B31] Kulik Á. Vida I. Fukazawa Y. Guetg N. Kasugai Y. Marker C. L. (2006). Compartment-dependent colocalization of Kir3.2-containing K+ channels and GABA_B_ receptors in hippocampal pyramidal cells. *J. Neurosci.* 26 4289–4297 10.1523/JNEUROSCI.4178-05.2006 PMC6673994 16624949

[B32] Kulik Á. Vida I. Luján R. Haas C. A. López-Bendito G. Shigemoto R. (2003). Subcellular localization of metabotropic GABA_B_ receptor subunits GABA_B_1a/b and GABA_B_2 in the rat hippocampus. *J. Neurosci.* 23 11026–11035. 14657159 10.1523/JNEUROSCI.23-35-11026.2003 PMC6741037

[B33] Lanthorn T. H. Cotman C. W. (1981). Baclofen selectively inhibits excitatory synaptic transmission in the hippocampus. *Brain Res.* 225 171–178 10.1016/0006-8993(81)90326-7 6271336

[B34] Larkum M. (2013). A cellular mechanism for cortical associations: an organizing principle for the cerebral cortex. *Trends Neurosci.* 36 141–151 10.1016/j.tins.2012.11.006 23273272

[B35] Longair M. H. Baker D. A. Armstrong J. D. (2011). Simple Neurite Tracer: open source software for reconstruction, visualization and analysis of neuronal processes. *Bioinformatics* 27 2453–2454 10.1093/bioinformatics/btr390 21727141

[B36] Lüscher C. Jan L. Y. Stoffel M. Malenka R. C. Nicoll R. A. (1997). G protein-coupled inwardly rectifying K+ channels (GIRKs) mediate postsynaptic but not presynaptic transmitter actions in hippocampal neurons. *Neuron* 19 687–695 10.1016/S0896-6273(00)80381-5 9331358

[B37] Magee J. C. Johnston D. (1997). A synaptically controlled, associative signal for Hebbian plasticity in hippocampal neurons. *Science* 275 209–213 10.1126/science.275.5297.209 8985013

[B38] Megías M. Emri Z. Freund T. F. Gulyás A. I. (2001). Total number and distribution of inhibitory and excitatory synapses on hippocampal CA1 pyramidal cells. *Neuroscience* 102 527–540 10.1016/S0306-4522(00)00496-6 11226691

[B39] Mott D. D. Bragdon A. C. Lewis D. V. Wilson W. A. (1989). Baclofen has a proepileptic effect in the rat dentate gyrus. *J. Pharmacol. Exp. Ther.* 249 721–725. 2543809

[B40] Mott D. D. Lewis D. V. Ferrari C. M. Wilson W. A. Swartzwelder H. S. (1990). Baclofen facilitates the development of long-term potentiation in the rat dentate gyrus. *Neurosci. Lett.* 113 222–226 10.1016/0304-3940(90)90307-U 2377319

[B41] Mott D. D. Li Q. Okazaki M. M. Turner D. A. Lewis D. V. (1999). GABA_B_-Receptor-mediated currents in interneurons of the dentate-hilus border. *J. Neurophysiol.* 82 1438–1450. 10482760 10.1152/jn.1999.82.3.1438

[B42] Nehring R. B. Horikawa H. P. M. El Far O. Kneussel M. Brandstätter J. H. Stamm S. (2000). The metabotropic GABA_B_ receptor directly interacts with the activating transcription factor 4. *J. Biol. Chem.* 275 35185–35191 10.1074/jbc.M002727200 10924501

[B43] Newberry N. R. Nicoll R. A. (1985). Comparison of the action of baclofen with gamma-aminobutyric acid on rat hippocampal pyramidal cells in vitro. *J. Physiol.* 360 161–185 10.1113/jphysiol.1985.sp015610 3989713 PMC1193454

[B44] Olah S. Fule M. Komlosi G. Varga C. Baldi R. Barzo P. (2009). Regulation of cortical microcircuits by unitary GABA-mediated volume transmission. *Nature* 461 1278–1281 10.1038/nature08503 19865171 PMC2771344

[B45] Otis T. S. De Koninck Y. Mody I. (1993). Characterization of synaptically elicited GABA_B_ responses using patch-clamp recordings in rat hippocampal slices. *J. Physiol.* 463 391–407 10.1113/jphysiol.1993.sp019600 8246190 PMC1175349

[B46] Otis T. S. Mody I. (1992). Differential activation of GABAA and GABA_B_ receptors by spontaneously released transmitter. *J. Neurophysiol.* 67 227–235. 1348084 10.1152/jn.1992.67.1.227

[B47] Palmer L. M. Schulz J. M. Murphy S. C. Ledergerber D. Murayama M. Larkum M. E. (2012). The cellular basis of GABA_B_-mediated interhemispheric inhibition. *Science* 335 989–993 10.1126/science.1217276 22363012

[B48] Pérez-Garci E. Larkum M. E. Nevian T. (2013). Inhibition of dendritic Ca2+ spikes by GABAB receptors in cortical pyramidal neurons is mediated by a direct Gi/o-βγ-subunit interaction with Cav1 channels. *J. Physiol.* 591 1599–1612 10.1113/jphysiol.2012.245464 23184512 PMC3624841

[B49] Pham T. M. Nurse S. Lacaille J.-C. (1998). Distinct GABA_B_ actions via synaptic and extrasynaptic receptors in rat hippocampus in vitro. *J. Neurophysiol.* 80 297–308. 9658051 10.1152/jn.1998.80.1.297

[B50] Ponce A. Bueno E. Kentros C. Vega-Saenz de Miera E. Chow A. Hillman D. (1996). G-protein-gated inward rectifier K+ channel proteins (GIRK1) are present in the soma and dendrites as well as in nerve terminals of specific neurons in the brain. *J. Neurosci.* 16 1990–2001. 8604043 10.1523/JNEUROSCI.16-06-01990.1996 PMC6578514

[B51] Price C. J. Cauli B. Kovacs E. R. Kulik A. Lambolez B. Shigemoto R. (2005). Neurogliaform Neurons Form a Novel Inhibitory Network in the Hippocampal CA1 Area. *J. Neurosci.* 25 6775–6786 10.1523/JNEUROSCI.1135-05.2005 16033887 PMC6725364

[B52] Price C. J. Scott R. Rusakov D. A. Capogna M. (2008). GABA_B_ receptor modulation of feedforward inhibition through hippocampal neurogliaform cells. *J. Neurosci.* 28 6974–6982 10.1523/JNEUROSCI.4673-07.2008 18596171 PMC2685170

[B53] Rial Verde E. Zayat L. Etchenique R. Yuste R. (2008). Photorelease of GABA with visible light using an inorganic caging group. *Front. Neural Circuits* 2:2 10.3389/neuro.04.002.2008 PMC2567106 18946542

[B54] Scanziani M. (2000). GABA spillover activates postsynaptic GABA_B_ receptors to control rhythmic hippocampal activity. *Neuron* 25 673–681 10.1016/S0896-6273(00)81069-7 10774734

[B55] Schmidt-Hieber C. Jonas P. Bischofberger J. (2007). Subthreshold dendritic signal processing and coincidence detection in dentate gyrus granule cells. *J. Neurosci.* 27 8430–8441 10.1523/JNEUROSCI.1787-07.2007 17670990 PMC6673070

[B56] Sloviter R. S. Ali-Akbarian L. Elliott R. C. Bowery B. J. Bowery N. G. (1999). Localization of GABA_B_ (R1) receptors in the rat hippocampus by immunocytochemistry and high resolution autoradiography, with specific reference to its localization in identified hippocampal interneuron subpopulations. *Neuropharmacology* 38 1707–1721 10.1016/S0028-3908(99)00132-X 10587087

[B57] Sodickson D. L. Bean B. P. (1996). GABA_B_ receptor-activated inwardly rectifying potassium current in dissociated hippocampal CA3 neurons. *J. Neurosci.* 16 6374–6385. 8815916 10.1523/JNEUROSCI.16-20-06374.1996 PMC6578909

[B58] Solís J. M. Nicoll R. A. (1992). Pharmacological characterization of GABA_B_-mediated responses in the CA1 region of the rat hippocampal slice. *J. Neurosci.* 12 3466–3472. 1326606 10.1523/JNEUROSCI.12-09-03466.1992 PMC6575740

[B59] Spruston N. Jaffe D. B. Williams S. H. Johnston D. (1993). Voltage- and space-clamp errors associated with the measurement of electrotonically remote synaptic events. *J. Neurophysiol.* 70 781–802. 8410172 10.1152/jn.1993.70.2.781

[B60] Stuart G. Spruston N. (1998). Determinants of voltage attenuation in neocortical pyramidal neuron dendrites. *J. Neurosci.* 18 3501–3510. 9570781 10.1523/JNEUROSCI.18-10-03501.1998 PMC6793161

[B61] Szilágyi T. De Schutter E. (2004). Effects of variability in anatomical reconstruction techniques on models of synaptic integration by dendrites: a comparison of three Internet archives. *Eur. J. Neurosci.* 19 1257–1266 10.1111/j.1460-9568.2004.03222.x 15016083

[B62] Takigawa T. Alzheimer C. (1999). G protein-activated inwardly rectifying K+ (GIRK) currents in dendrites of rat neocortical pyramidal cells. *J. Physiol.* 517 385–390 10.1111/j.1469-7793.1999.0385t.x 10332089 PMC2269339

[B63] Tanaka J. Matsuzaki M. Tarusawa E. Momiyama A. Molnar E. Kasai H. (2005). Number and density of AMPA receptors in single synapses in immature cerebellum. *J. Neurosci.* 25 799–807 10.1523/JNEUROSCI.4256-04.2005 15673659 PMC6725634

[B64] Tao W. Higgs M. H. Spain W. J. Ransom C. B. (2013). Postsynaptic GABA_B_ receptors enhance extrasynaptic GABAA receptor function in dentate gyrus granule cells. *J. Neurosci.* 33 3738–3743 10.1523/JNEUROSCI.4829-12.2013 23447585 PMC6619291

[B65] Uematsu M. Hirai Y. Karube F. Ebihara S. Kato M. Abe K. (2008). Quantitative chemical composition of cortical GABAergic neurons revealed in transgenic venus-expressing rats. *Cereb. Cortex* 18 315–330 10.1093/cercor/bhm056 17517679

[B66] White J. H. McIllhinney R. A. J. Wise A. Ciruela F. Chan W.-Y. Emson P. C. (2000). The GABA_B_ receptor interacts directly with the related transcription factors CREB2 and ATFx. *Proc. Natl. Acad. Sci. U.S.A.* 97 13967–13972 10.1073/pnas.240452197 11087824 PMC17684

[B67] Williams S. Lacaille J.-C. (1992). GABA_B_ receptor-mediated inhibitory postsynaptic potentials evoked by electrical stimulation and by glutamate stimulation of interneurons in Stratum lacunosum-moleculare in hippocampal CA1 pyramidal cells in vitro. *Synapse* 11 249–258 10.1002/syn.890110309 1353275

